# Glutamic Acid Decarboxylase-Derived Epitopes with Specific Domains Expand CD4^+^CD25^+^ Regulatory T Cells

**DOI:** 10.1371/journal.pone.0007034

**Published:** 2009-09-13

**Authors:** Guojiang Chen, Gencheng Han, Jiannan Feng, Jianan Wang, Renxi Wang, Ruonan Xu, Beifen Shen, Jiahua Qian, Yan Li

**Affiliations:** 1 Department of Molecular Immunology, Institute of Basic Medical Sciences, Beijing, P.R. China; 2 National Cancer Institute, Vaccine branch, Bethesda, Maryland, United States of America; Singapore Immunology Network, Singapore

## Abstract

**Background:**

CD4^+^CD25^+^ regulatory T cell (Treg)-based immunotherapy is considered a promising regimen for controlling the progression of autoimmune diabetes. In this study, we tested the hypothesis that the therapeutic effects of Tregs in response to the antigenic epitope stimulation depend on the structural properties of the epitopes used.

**Methodology/Principal Findings:**

Splenic lymphocytes from nonobese diabetic (NOD) mice were stimulated with different glutamic acid decarboxylase (GAD)-derived epitopes for 7–10 days and the frequency and function of Tregs was analyzed. We found that, although all expanded Tregs showed suppressive functions *in vitro*, only p524 (GAD524–538)-expanded CD4^+^CD25^+^ T cells inhibited diabetes development in the co-transfer models, while p509 (GAD509–528)- or p530 (GAD530–543)-expanded CD4^+^CD25^+^ T cells had no such effects. Using computer-guided molecular modeling and docking methods, the differences in structural characteristics of these epitopes and the interaction mode (including binding energy and identified domains in the epitopes) between the above-mentioned epitopes and MHC class II I-A^g7^ were analyzed. The theoretical results showed that the epitope p524, which induced protective Tregs, possessed negative surface-electrostatic potential and bound two chains of MHC class II I-A^g7^, while the epitopes p509 and p530 which had no such ability exhibited positive surface-electrostatic potential and bound one chain of I-A^g7^. Furthermore, p524 bound to I-A^g7^ more stably than p509 and p530. Of importance, we hypothesized and subsequently confirmed experimentally that the epitope (GAD570–585, p570), which displayed similar characteristics to p524, was a protective epitope by showing that p570-expanded CD4^+^CD25^+^ T cells suppressed the onset of diabetes in NOD mice.

**Conclusions/Significance:**

These data suggest that molecular modeling-based structural analysis of epitopes may be an instrumental tool for prediction of protective epitopes to expand functional Tregs.

## Introduction

Glutamic acid decarboxylase (GAD65), consisting of 585 amino acids, has been shown to be the first of several β cell antigens to be recognized by T cells from nonobese diabetic (NOD) mice and patients with type 1 diabetes [1]–[3]. Its importance in the pathogenesis of autoimmune diabetes is indicated by the following evidence: 1. T cell responses against GAD65 can be detected in young NOD mice and in humans at risk of type 1 diabetes [4], [5]; 2. β cell-specific suppression of GAD65 expression in antisense GAD65 transgenic NOD mice leads to complete prevention of type 1 diabetes and blocks the generation of diabetogenic T cells [6]. These findings suggest that modulation of GAD65 autoimmunity can influence the development of type 1 diabetes in NOD mice.

It is known that GAD65 contains dozens of epitopes which are specifically recognized by T cells as peptide epitope-MHC complexes on the surface of target cells thereby directing T cell function [7]. Indeed, pretreatment of prediabetic NOD mice with GAD65 or some of its peptide epitopes efficiently delays the onset of type 1 diabetes [8]. Strikingly, Quinn et al. [9] found the presence of two kinds of epitopes (named as effector and regulatory or protective epitopes) in GAD65 which were completely different in their mediation of T cell function and the pathogenesis of diabetes. Although many factors such as the mature state of antigen processing cells (APC) contribute to determining whether an epitope is an effector or regulatory epitope, the physical-chemical properties of epitopes may have important effects on their function. Differential structural properties of epitopes probably lead to divergent conformation of MHC class II molecules bound to the corresponding epitopes and ultimately to differential T cell functional activation. Peptide epitope immunization in NOD mice is associated with the induction of Tregs that secrete IL-10 and/or transforming growth factor (TGF)-β [10], [11]. These cells have been shown to be pivotal for immune homeostasis and protection against autoimmunity [12]. Recently, it is reported that Tregs can be expanded by dendritic cells bearing an autoantigenic peptide from pancreatic β cells [13]. Thus, we postulate that there is a difference between effector and regulatory epitopes in their ability to expand Tregs *in vitro*.

Previous research in our lab has demonstrated that a single immunization of GAD_500–585_ (a peptide of GAD65) in young NOD mice remarkably reduced pancreatic insulitis and efficiently prevented the development of overt type 1 diabetes [14]. In fact, GAD_500–585_ is composed of multiple epitopes such as 509–528, 524–538 and 530–543, which are important autoantigenic peptides for the development of type 1 diabetes [1], [9]. In this study, the potential capacity of the above three peptide epitopes to expand CD4^+^CD25^+^ T cells *ex vivo* was investigated and the *in vivo* suppressive effects of these expanded CD4^+^CD25^+^ T cells were then examined. Furthermore, a potential relationship between the structure and function of these epitopes was analyzed by molecular modeling/docking methods and a novel epitope p570 in GAD_500–585_ which induced protective Tregs was presented.

## Materials and Methods

### Mice

NOD, and NOD.scid female mice were obtained from the Jackson Laboratory and bred in our facilities under specific pathogen-free conditions. Care, use and treatment of mice in this study was in strict agreement with guidelines in the care and use of laboratory animal manual set out by the Institute of Basic Medical Sciences. Diabetes monitoring was performed on a weekly measurement basis. The female NOD mice develop overt diabetes at the age of 12 wks and the incidence reaches 80% by 25 wks of age.

### Epitope analysis and synthesis

The continuous epitopes of GAD_500–585_ were determined based on its primary structure using the BIOSUN software [http://www.biosun.org.cn/biosun/intro.htm, ref. 15] bearing in mind its hydrophilic-hydrophobic properties [16], secondary structure [17], antigenicity [18] and accessibility of the residues [19]. In order to understand the relationship between sequence and structure in greater depth, the 3-D structure of GAD_500–585_ was constructed using the homology modeling method (InsightII software (2005), Accelrys, San Diego, CA) based on the crystal structure of GAD65. To optimize the packing interactions through protein side-chain repacking, the 3-D modeled structure of GAD_500–585_ was minimized with molecular mechanics method under Consistent Valence Force Field (CVFF) and Gromos96 force field, respectively. Atomic charges were taken from the corresponding force field package. Cross-terms were not used and energy convergence was considered to have been obtained when the maximum derivative reached 4.2 J/Å by the conjugate gradients method.

The surface electrostatic potential was calculated based on the stable 3-D structure of GAD_500–585_, using the DELPHI program (InsightII software (2005), Accelrys, San Diego, CA). The charges of the ionized groups were assigned assuming standard amino acid protonation state at pH 7. Hisidines were considered in the neutral form. The CVFF partial atomic charges were used and the dielectric constant of GAD_500–585_ and solvent (i.e. water) were set to 2 and 80, respectively. An ion exclusion layer was added to the solutions and extended 10 Å beyond the molecular surfaces. The non-linear Poisson–Boltzmann equation was solved in the finite difference approximation, and the numerical calculations of the potential were iterated to convergence, which was defined as the point at which the potential changes less than 10^−4^ kT/emol between successive iterations.

GAD peptides p509, p524, p530 and p570 were synthesized at Genemed Synthesis, and were brought to >90% purity using reverse phase HPLC. Peptide purity was determined by capillary electrophoresis, and the amino acid composition of the peptides was verified by mass spectrometry. Ovalbumin (OVA) was purchased from Sigma-Aldrich (USA).

### Molecular docking

3-D structures of the above-mentioned peptides were constructed using the *ab initio* modeling method [20], [21]. Firstly, secondary structures of the peptides were predicted and dihedral angles were determined. 3-D original structures of the peptides were then modeled. Finally, the stable structures of the peptides were obtained under CVFF using molecular mechanics optimization.

Using the 3-D crystal structure of the MHC II molecule and the modeling structure of the GAD65 peptide in complex with murine MHC class II I-A^g7^ (PDB code: 1es0) as templates, bearing in mind Van der Waals and intermolecular hydrogen bonding interactions, the 3-D structures of the peptides in complex with the MHC class II molecule were constructed and optimized with the molecular docking method.

In order to minimize steric clashes and ensure correct interaction non-bond distances, angles and hydrogen bonds after molecular docking, the interaction domains between the peptides and MHC class II molecule were subjected to 5000 steps of energy minimization, while the remainder was held fixed in position. To avoid the conformation of the interaction domains trapped in a local potential energy minimum, residues at the base of the interaction domains were held fixed while the remainder of the interaction domains was subjected to simulated heating and molecular dynamics at elevated temperatures followed by slow cooling to a low energy conformation. The interaction domain residues were initially assigned a temperature of 300K and slowly heated to 500K in increments of 25K, with 50 dynamics steps at each temperature using a time step of 1 fs. The structure was similarly heated to 1000, 2000, 3000 and 4000K. At each temperature the interaction domains were subjected to a 100 ps dynamics run followed by slowing to 300K, and two series of minimizations, first for 500 steps, then 3000 steps.

### Flow cytometry

Antibodies used for flow cytometry were as follows: PE-labeled mAbs to CD4 (GK1.5), LAG-3 (C9B7W), CTLA-4 (UC10-4F10-11) and ICOS (7E.17G9) were purchased from BD Pharmingen, and FITC-labeled anti-CD4 (GK1.5) and PE-Cy5-labeled anti-CD25 (PC61) mAbs, PE-conjugated mAbs against GITR (DTA-1), and Foxp3 (FJK-16s) were purchased from *e*Bioscience. Cells were stained in PBS containing 2% heat-inactivated FCS and 0.2% sodium azide, and fixed using PBS containing 1% paraformaldehyde. For intracellular staining, cells were first stained with Abs for 30 min and then fixed for 20 min with 1 ml fixation buffer (Fix & Perm cell permeabilization kit; *e*Bioscience). After washing, the fixed cells were incubated with Abs for 30 min. Data collection and analysis were performed on a FACS Calibur flow cytometer using CellQuest software (Becton Dickinson).

### *In vitro* expansion of epitope-specific T cells

Splenic lymphocytes were isolated from 4–8 wk old female NOD mice and cultured with different peptide epitopes or OVA control (50 ng/ml) in RPMI-1640 medium, which consisted of 1% horse serum (Hyclone), nonessential amino acids, 0.5 mM sodium pyruvate, 5 mM Hepes, 1 µM β-mercaptoethanol. The cultures were monitored daily and maintained at 1−1.5×10^6^/ml by diluting with complete medium for 7–10 days.

### Purification of CD4^+^CD25^+^ T cells and *in vitro* suppression assays

Purification of CD4^+^CD25^+^ T cells by MACS (Miltenyi Biotec, Germany) was as described previously [22]. In brief, CD4^+^ T cells were isolated by depletion of magnetically labeled non-CD4^+^ T cells. CD4^+^ T cells labeled with PE-conjugated anti-CD25 were then magnetically labeled with anti-PE microbeads. The magnetically labeled cells were passed through a column placed in the magnetic field of a MACS separator and separated into CD4^+^CD25^+^ and CD4^+^CD25^−^ T cells (purity>96%).

For suppression assays, graded numbers of expanded or freshly purified CD4^+^CD25^+^ T cells were added to 50,000 CD4^+^ T cells stimulated with 50,000 irradiated splenic APCs (2,000 rads) and 1 µg/ml anti-CD3 in U-bottomed 96-well plates. CD4^+^ T cell cultures without CD4^+^CD25^+^ T cells were stimulated in the same manner as positive controls. The cultures were maintained at 37°C for a total of 72 h and cell proliferation was measured by incorporation of [^3^H]thymidine (1 µCi/well) during the final 16 h of culture.

### Adoptive transfer

Epitope-expanded CD4^+^CD25^+^ T cells were purified as described above and injected intravenously (at different cell numbers, as indicated) into 4–8 wk old NOD.scid mice along with diabetogenic splenocytes isolated from acutely diabetic NOD mice. Recipients only receiving diabetogenic splenocytes were regarded as positive controls. In some experiments, expanded CD4^+^CD25^+^ T cells (1−3×10^6^) were transfused into 4–6 wk old NOD mice. In combination of adoptive transfer, some mice received intraperitoneally antibodies to IL-10/TGF-β or isotype IgG (all from R&D systems), respectively. The regimens of antibodies to IL-10 and TGF-β administration were following: a dose of 0.5 mg/mouse on days −1, 0, 2, 5, then every 5 days for anti-IL-10; a dose of 1 mg/mouse on days 0, 3, 5, 8, then every 5 days for anti-TGF-β. Nonfasting blood glucose levels in recipient mice were monitored using a MediSense glucometer (Abbott Laboratories) and blood glucose ≥300 mg/dl was considered diabetic.

### ELISA of cytokine production

After splenocytes were incubated as described above, supernatants were harvested and IL-2, IL-10 and TGF-β production was assayed by sandwich ELISA. The ELISA kits used in this study were purchased from BD Pharmingen.

### Statistical analysis

The Kaplan-Meier method was used to compare diabetes-free survival and incidence. The Student's *t*-test was used to compare mean values. Values of *P*<0.05 were considered significant.

## Results

GAD_500–585_-derived peptide epitope stimulation expands CD4^+^CD25^+^Foxp3^+^ T cells from NOD mice

**Figure 1:**
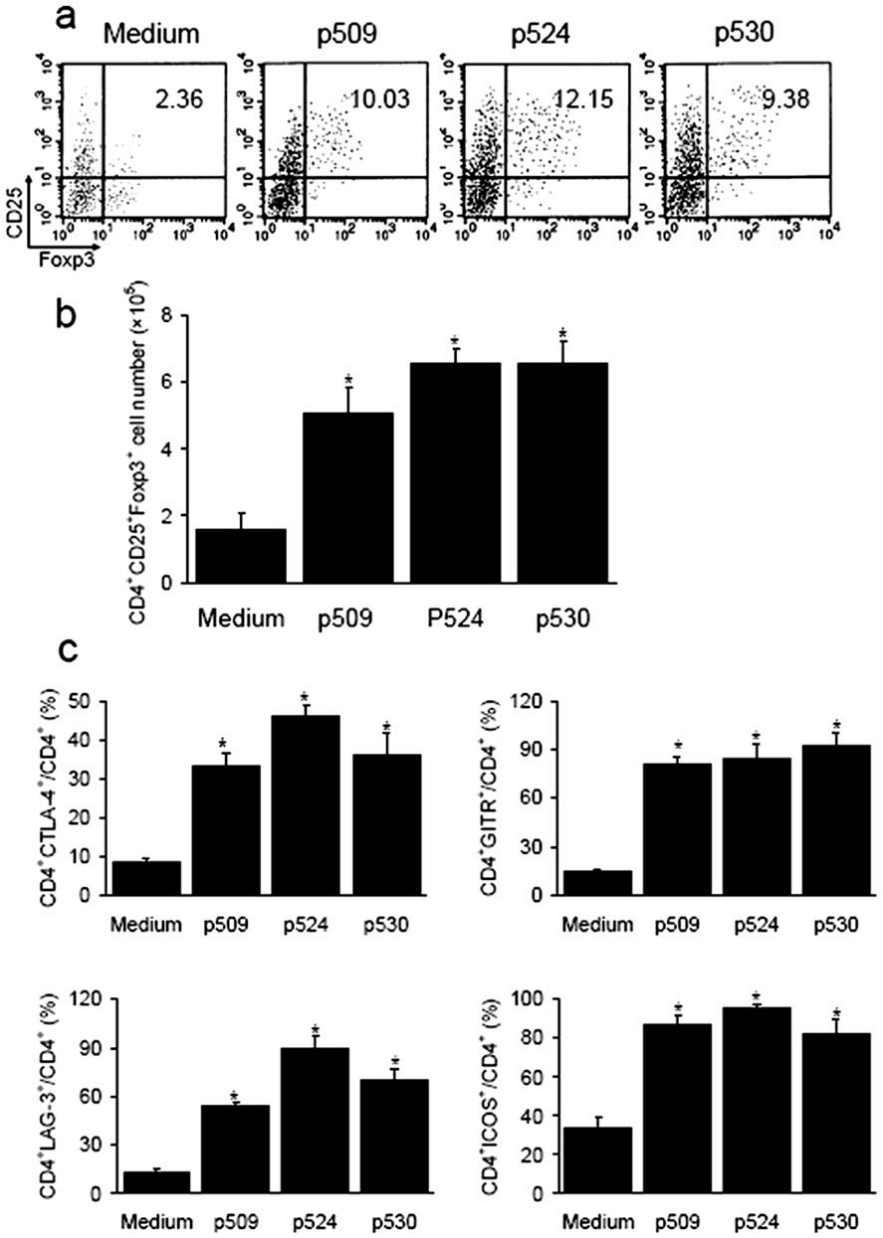
*In vitro* expansion of CD4^+^CD25^+^ Foxp3^+^ T cells by peptide epitope stimulation. Splenic lymphocytes were isolated from one NOD mice (total number of mice was 6) and cultured with different peptide epitopes (50 ng/ml) for 7-10 d. Cells were collected and analysed by flow cytometry. (a) The percentages of CD25^+^Foxp3^+^ within CD4^+^cells were presented. (b) Total number of CD4^+^CD25^+^Foxp3^+^ T cells in each group after expansion. Data collected from three independent experiments are shown. (c) Expression of Treg-related phenotypes (CTLA-4, GITR, ICOS, LAG-3) in CD4 T cells from each group. As controls, OVA (50 ng/ml) stimulation could not expand CD4^+^CD25^+^Foxp3^+^ T cells and up-regulate the expression of Treg-related molecules (data not shown). Data are shown as means±SD, n = 5–6 per group. *, *P*<0.05 compared with controls.

Steinman and colleagues reported that β-cell autoantigenic peptide-bearing dendritic cells (DCs) expanded functionally competent Tregs which subsequently suppressed diabetes development in an antigen-specific manner [13]. Here, in order to determine whether known autoantigenic epitopes of GAD_500-585_, such as p509, p524 and p530, could also expand suppressive Tregs, splenic lymphocytes from autoimmune diabetes-prone NOD mice were exposed to stimulation from different peptide epitopes for 7–10 days. Since Foxp3 is known to be a master regulator for CD4^+^CD25^+^ Treg development [23]–[25], CD4^+^CD25^+^Foxp3^+^ T cells were examined. We found that the percentage and number of these cells increased 2–10 fold in response to stimulation with the above peptide epitopes compared to controls (**Figure 1a and 1b**). In addition, Treg-associated molecules, such as cytotoxic T lymphocyte-associated antigen (CTLA)-4 [26], glucocorticoid-induced TNFR-related protein (GITR) [27], inducible costimulator (ICOS) [28] and lymphocyte activation gene (LAG)-3 [29], were also up-regulated compared to controls (**Figure 1c**).

**Figure 2:**
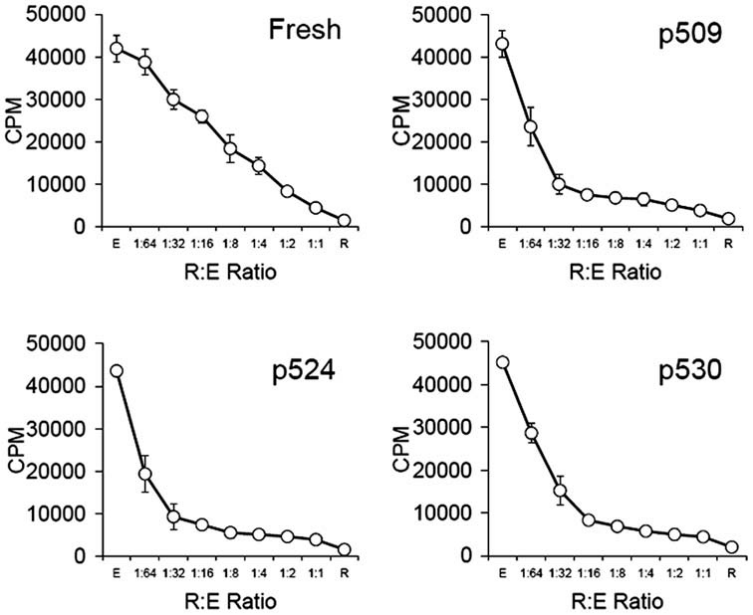
*In vitro* suppression by expanded CD4^+^CD25^+^ T cells. CD4^+^CD25^+^ T cells (R) with or without peptide epitope stimulation were compared for their ability to suppress the proliferation of CD4^+^ effector T cells (E) stimulated with anti-CD3 and irradiated APC at graded ratios. The proliferation was measured by [^3^H]thymidine uptake. Results are shown as means±SD. The data were collected from six to eight separate experiments.

To further determine whether the peptide epitope-expanded CD4^+^CD25^+^ T cells were functionally suppressive, these expanded cells were examined for their ability to suppress CD4^+^ T effector cells (Teffs) from diabetic NOD mice. Tregs and Teffs were co-cultured in the presence of anti-CD3 and irradiated APC at a titred ratio. Results showed that the expanded CD4^+^CD25^+^ T cells efficiently suppressed proliferation of effector cells. This suppressive effect was more potent than for freshly isolated Tregs, as suppression was routinely observed at a Treg/Teff ratio of <1∶32 (**Figure 2**). These results show that suppressive GAD_500–585_ epitope-expanded CD4^+^CD25^+^ T cells can be obtained *in vitro* by addition of peptide epitopes to pulse splenic APC.

**Figure 3:**
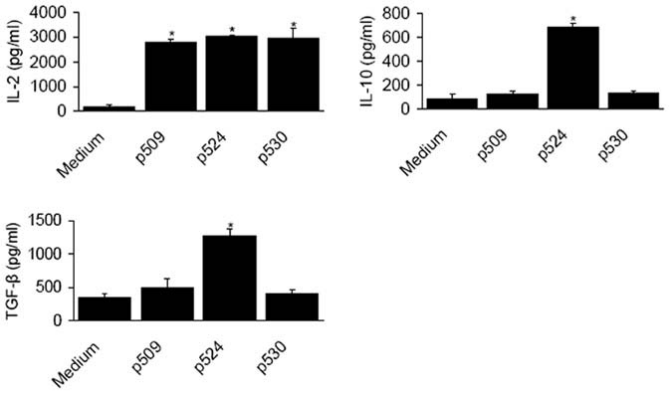
Cytokine production of expanded CD4^+^CD25^+^ T cells after peptide epitope stimulation. After 7–10d incubation with different peptide epitopes, the IL-2, IL-10 and TGF-β production of expanded CD4^+^CD25^+^ T cells was determined by sandwich ELISA. Results are shown as means±SD. The data were collected from four to six separate experiments. *, *P*<0.05 compared with controls.

Next, to determine the mechanisms underlying inhibitory effects of the expanded CD4^+^CD25^+^ T cells, we examined their cytokine profiles in response to differential epitope stimulation. The CD4^+^CD25^+^ T cells from each group secreted high amounts of IL-2 (**Figure 3a**), in line with the hypothesis that IL-2 is a key cytokine for expansion and survival of Tregs [30], [31]. Strikingly, CD4^+^CD25^+^ T cells produced more IL-10 and TGF-β in response to p524 stimulation than in response to p509 or p530 stimulation (**Figure 3b and 3c**), indicating that p524-expanded CD4^+^CD25^+^ T cells may be more competent. We also examined IL-4 and IFN-γ production, but no significant differences were found between the p509, p524 and p530 stimulation groups (data not shown).

*In vitro*-expanded **CD4^+^CD25^+^ T cells** suppress adoptive transfer of diabetes *in vivo*

**Figure 4:**
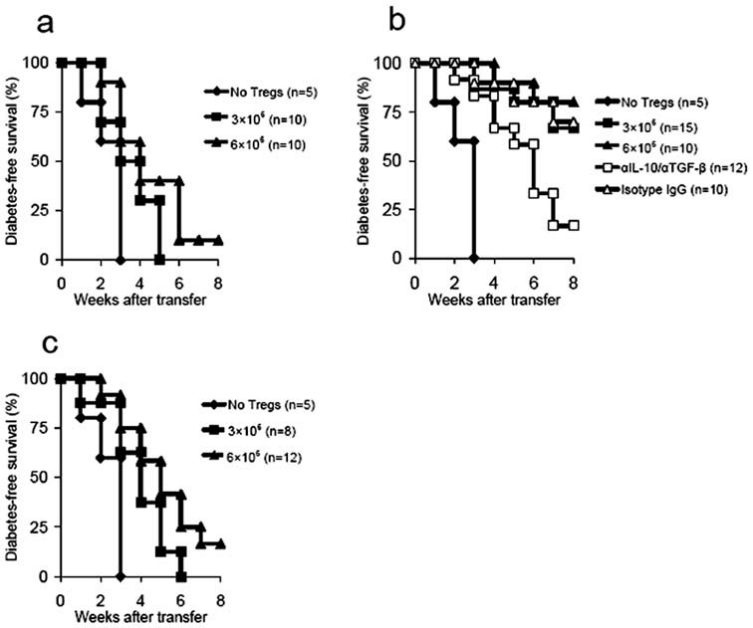
P524-expanded CD4^+^CD25^+^ T cells prevented transfer of diabetes. Diabetic splenocytes (1×10^7^) were co-transferred with or without CD4^+^CD25^+^ T cells expanded by p509 (a), p524 (b), or p530 (c), to 4–8 wk old NOD.scid mice at the numbers indicated. In some cases, NOD.scid recipients receiving 6×10^6^ p524-expanded CD4^+^CD25^+^ T cells were injected with neutralizing antibodies to IL-10/TGF-β according to the regimens described in Materials and Methods. Blood glucose was monitored for up to 8 wks. Differences in blood glucose levels between diabetic spleens alone and diabetic spleens plus 3×10^6^ p524-expanded CD4^+^CD25^+^ T cells were significant (P = 0.003), as were differences between diabetic spleens alone and diabetic spleens plus 6×10^6^ p524-expanded CD4^+^CD25^+^ T cells (P = 0.002). Cumulative data from three experiments are shown. The number of mice in each group is indicated in parentheses.

Using a co-transfer model, we subsequently examined the ability of epitope-expanded CD4^+^CD25^+^ T cells to suppress diabetes when co-transferred with diabetogenic T cells (1×10^7^ cells/mouse) into NOD.scid mice. Interestingly, we found that p524-expanded CD4^+^CD25^+^ T cells (3×10^6^) effectively blocked diabetes transfer, whereas the other two types of epitope-expanded CD4^+^CD25^+^ T cells did not show such protective effects, even when much higher numbers of expanded CD4^+^CD25^+^ T cells were used (**Figure 4**). Furthermore, neutralizing antibodies to IL-10/TGF-β were administrated to evaluate the role of IL-10/TGF-β in p524-expanded CD4^+^CD25^+^ T cell-mediated prevention from diabetes transfer. The results showed that blockade of IL-10/TGF-β bioactivities in vivo dramatically abrogated the suppressive effects of p524-expanded CD4^+^CD25^+^ T cells on adoptively transfer of diabetes (**Figure 4b**). Intriguingly, the neutralization of one of these two cytokines (IL-10 or TGF-β) just had minor effects on Treg-mediated blockade of diabetes development (data not shown), indicating a complementary role of IL-10 or TGF-β in mediating Treg protective effects when one cytokine was removed. We also examined the ability of polyclonal CD4^+^CD25^+^ T cells expanded by anti-CD3 plus IL-2 for inhibition of diabetes transfer. Consistent with a previous report [32], our results showed that high numbers of these CD4^+^CD25^+^ T cells (6×10^6^) were insufficient to prevent diabetes (data not shown).

### *In vitro*-expanded CD4^+^CD25^+^ T cells delay or suppress the onset of diabetes in prediabetic NOD mice

**Figure 5:**
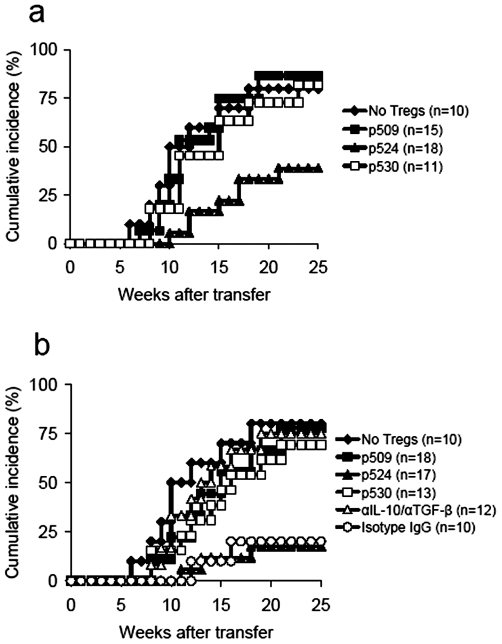
P524-expanded CD4^+^CD25^+^ T cells prevented/delayed the onset of diabetes in autoimmune diabetes-prone NOD mice. 4–6 wk old NOD mice were transfused with CD4^+^CD25^+^ T cells (either 1×10^6^ (a) or 3×10^6^ (b) per mouse) expanded by p509, p524 or p530. Untreated littermates were used as controls. In some cases, NOD recipients receiving 3×10^6^ p524-expanded CD4^+^CD25^+^ T cells were injected with neutralizing antibodies to IL-10/TGF-β according to the regimens described in Materials and Methods. Development of diabetes was monitored for over 25 wks and the cumulative incidence of diabetes was shown from two experiments. The difference between untreated controls and mice treated with 1×10^6^ p524-expanded CD4^+^CD25^+^ T cells was significant (P = 0.0036), as was that between untreated controls and mice treated with 3×10^6^ p524-expanded CD4^+^CD25^+^ T cells (P = 0.0015). The number of mice in each group is indicated in parentheses.

Since the immunoregulatory activity of Tregs based on adoptive transfer models that take advantage of lymphopenic settings to enhance Treg proliferation can be explained by active regulation or a side effect of competition for “space” [33], [34], we further investigated the diabetes-suppressive function of Tregs in a nonlymphopenic autoimmune diabetes-prone NOD mouse model. Previous studies have shown that NOD mice are deficient in Treg number and function [35]–[37] and that intravenous transfer of β cell-specific *ex vivo* expanded Tregs into NOD mice actively prevents diabetes [38], [39]. Thus, 4–6 wk old NOD mice were injected with 1−3×10^6^ CD4^+^CD25^+^ T cells and subsequently monitored for diabetes. Transfer of as few as 1×10^6^ p524-expanded CD4^+^CD25^+^ T cells per mouse markedly prevented/delayed the development of diabetes for as long as 25 wk after transfer (**Figure 5a**), whereas transfer of similar or even 3-fold greater numbers of p509 or p530-expanded CD4^+^CD25^+^ T cells only had a slight preventive effect on diabetes incidence (**Figure 5a and 5b**). In this setting, we also examined whether IL-10/TGF-β cytokines played an essential role in p524-expanded CD4^+^CD25^+^ T cell-mediated delayed development of diabetes. The findings showed that neutralization of IL-10/TGF-β in vivo significantly abolished the protective effects of these cells (**Figure 5b**), indicating that the immunoregulatory function of p524-expanded CD4^+^CD25^+^ T cells was partially attributed to high amounts of IL-10/TGF-β produced by these cells. Taken together, these results suggested that Treg-mediated inhibition of diabetes development is epitope-specific.

### GAD_500–585_ epitope structure-function study

**Figure 6:**
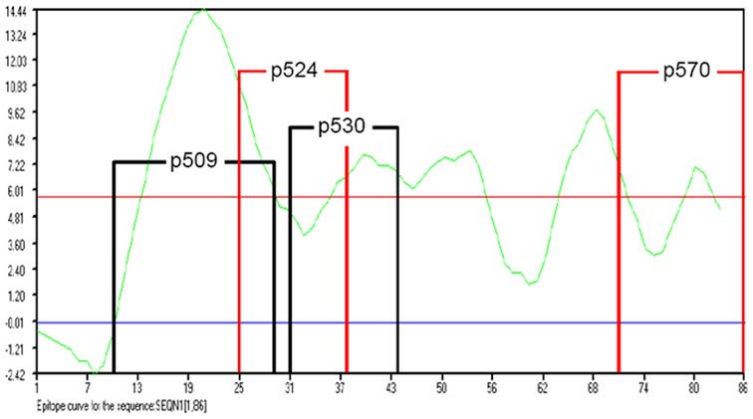
Epitopes in GAD_500–585_ were predicted using BIOSUN software bearing in mind sequence properties, the hydrophobic-hydrophilic map, secondary structure, antigenicity and accessibility. The X-coordinate denoted the sequence location in GAD500–585, of which the first value was the NO. 500 residue in GAD65 and the rest was deduced by analogy. The Y-coordinate denoted the statistical value from the sequence properties (including the hydrophobic-hydrophilic map, secondary structure, antigenicity and accessibility). The curve peak indicated the most possible key residue to form epitope. The region up the red line denoted the possible epitopes, and the region down the blue line denoted the impossible epitopes. The region between the blue and red lines indicated uncertain/potential epitopes. The peptides used in our study are marked as p509, p524, p530 and p570. The beginning and end points of the peptides are marked as vertical lines.

To determine whether this distinction in the Treg-expanding function of these epitopes was related to differences in their structural properties, physical and chemical features of p509, p524 and p530 were studied by surface property analysis and molecular modeling methods. The continuous epitopes of the GAD_500–585_ sequence were analyzed using BIOSUN software. As shown in **Figure 6**, peaks of the analyzed curve denoted the center of the continuous epitopes and peptides 509–528, 532–545, 530–543, 550–556, 562–574, and 570–584 appear to be the main epitopes of GAD_500–585_. In the above experiments, the epitopes p509 and p530 could be identified clearly, however p524 (GAD524–538) appeared to include two potential epitopes (i.e. the C-terminal of p509 and the N-terminal of p532). The predicted epitopes p509 and p530 were composed of one core epitope, while the epitope p524 was made up of two core epitopes. Our results are in line with a recent report that p524 and p530 share one core and that flanking residues are critical for their function [40]. Taken together, these results show a potential difference between p524, and p509 as well as p530.

**Figure 7:**
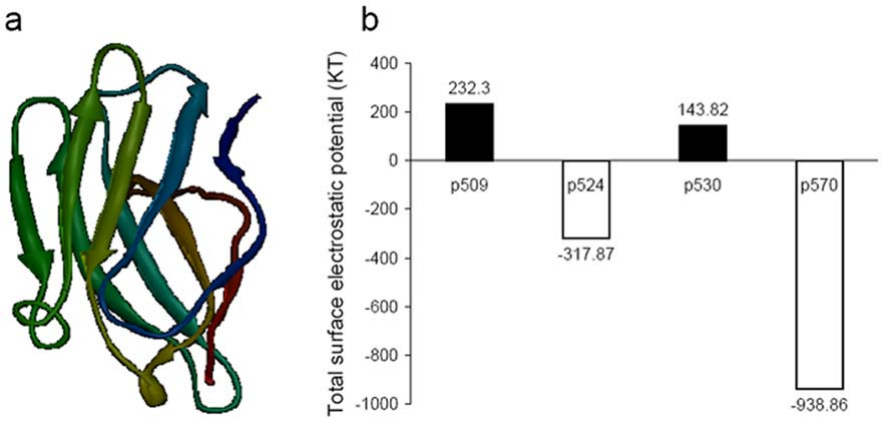
3-D structure and surface properties of GAD_500–585_. (a) 3-D modeling structure of GAD_500–585_, derived from the crystal structure of GAD65 using a computer modeling homology method and optimized using molecular mechanism and molecular dynamics methods under a CVFF forcefield, was shown as ribbon map and the secondary structure (composed of beta sheets and turns) was analyzed. (b) Surface electrostatic potential of the peptides p509, p524, p530, p570. The surface electrostatic potential of the residues in GAD_500–585_ was calculated using the DELPHI program, and the total surface electrostatic potential distribution of the peptides was summed. The positive value in Y-coordinate denoted the peptides possessed the positive surface electrostatic potential, and vice versa.

To understand more deeply the structure-function relationship of the epitopes, the 3-D structure of GAD_500–585_ was modeled and optimized based on the crystal structure of GAD from the PDB database (PDB code: 2okk) and the predicted secondary structure of GAD_500–585_ (data not shown), as shown in **Figure 7a**. It can be seen that GAD_500–585_ is composed of three alpha helices and three beta sheets. Due to the presence of five proline residues, the 3-D structure of GAD_500–585_ is compact. Our computer-modeled 3-D structure of GAD_500–585_ was very similar to that in the original protein structure of GAD, and the RMSD (Root Mean Square Distance) value of main chain carbon atoms between GAD_500–585_ and GAD was 0.0464 nm.

Furthermore, according to the modeling 3-D structure of GAD_500–585_, the surface electrostatic potential distribution of GAD_500–585_ was analyzed using DELPHI program. The calculated values are shown in **Figure 7b**. Results showed that fragments p509 and p530 possessed positive surface electrostatic potential, while fragments p524 and p570 possessed negative surface electrostatic potential. The surface electrostatic potential difference of the peptides might be related to their bio-function. The peptides p509 and p530 might possess the similar function while p524 and p570 had the similar abilities.

In summary, together with the experimental results, the conclusion is made that differences in the functions of p524, p509 and p530 in expanding competent Tregs may be due to differences in their structural properties.

### The structural mode of interaction with MHC II varies with peptide epitope

**Figure 8:**
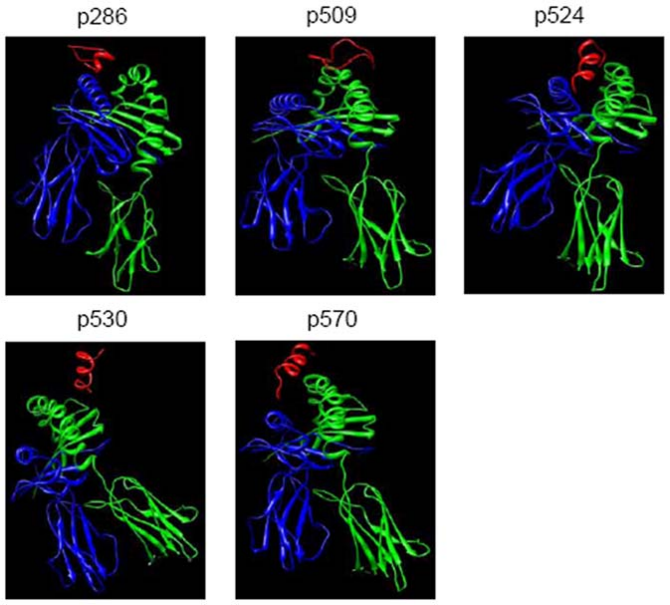
3-D optimized structures of the peptides (p286, p509, p524, p530 and p570) in complex with MHC class II I-A^g7^ using molecular docking and molecular dynamics methods. The red ribbon denoted the peptides and the blue and green ribbons denoted the dimer of the MHC class II I-A^g7^. The peptides p509 and p530 bound monomer of the MHC class II I-A^g7^, while the peptides p524 and p570 bound the core domain of the dimmer similar to the peptide p286.

The 3-D structures of the peptide epitopes p509, p524, p530 and p570 were modeled using secondary structure prediction and *ab initio* modeling methods, and 3-D structures of the interaction of peptide epitopes with I-A^g7^ were constructed by molecular docking methods, according to the 3-D crystal structure of MHC II I-A^g7^ in complex with p207 (PDB code: 1es0). Optimized 3-D structures are shown in **Figure 8**. The peptide p286 from GAD65 (286–300), whose transgenic expression delayed the onset of diabetes [41], was also analyzed by way of comparison. Results showed that p524 and p570 possessed a similar binding pattern to that of I-A^g7^ with p286. The two peptides interacted with the middle of the I-A^g7^ chain A and B. In contrast, peptides p509 and p530 interacted with I-A^g7^ chain B, but not with chain A.

Interaction energies of p286, p509, p524, p530 and p570 in complex with I-A^g7^ were calculated based on 3-D modeling structures. As shown in **Table 1**, the energy of p524 binding to I-A^g7^ was markedly lower than that of p509 and p530, but comparable to that of p286 and p570. To understand the mode of interaction between the peptides and I-A^g7^ more fully, the domains in the peptide sequence were identified by computer graphics technology and distance geometry methods and were shown in **Table 2**.

**Table 1:** The interaction energy (KJ/mol) between the indicated peptides and I-A^g7^.

The complex of the peptides and I-A^g7^	The interaction energy (KJ/mol)		
	Van Der Waals	Electrostatic	Total energy
p286-I-A^g7^	−75.45	−75.74	−151.19
p509-I-A^g7^	−36.37	−43.97	−80.34
p524-I-A^g7^	−84.94	−78.63	−163.57
p530-I-A^g7^	−31.43	−37.62	−69.05
p570-I-A^g7^	−69.10	−107.13	−176.23

Interaction energies were calculated under CVFF based on the 3-D theoretical structure of the peptides in complex with MHC class II I-A^g7^. The theoretical interaction energy was composed of Van der Waals interactions and electrostatic potential.

**Table 2:** The identified domain in I-A^g7^ by the indicated peptides.

The complex of the peptides and I-A^g7^	The identified domains in I-A^g7^
p286-I-A^g7^	A55, A57, A61, A67, B60, B64, B66, B70
p509-I-A^g7^	B76, B77, B81, B83
p524-I-A^g7^	A55, A57, A61, B69, B70, B73
p530-I-A^g7^	B21, B22, B76, B81, B83
p570-I-A^g7^	A53, A55, A57, A61, B64, B69, B70, B73

Interaction domains were determined using computer graphic technology and distance geometry methods based on the 3-D structures of the peptides in complex with MHC class II I-A^g7^.

Overall, considering differences in structures of the peptide-MHC class II complexes, interaction energies and identified domains, we concluded that peptides p524 and p570 possessed similar structures to p286, while peptides p509 and p530 were significantly different from the above peptides. Thus, we hypothesized that p570 might be a protective epitope.

P570 stimulation expands CD4^+^CD25^+^Foxp3^+^ T cells from NOD mice

**Figure 9:**
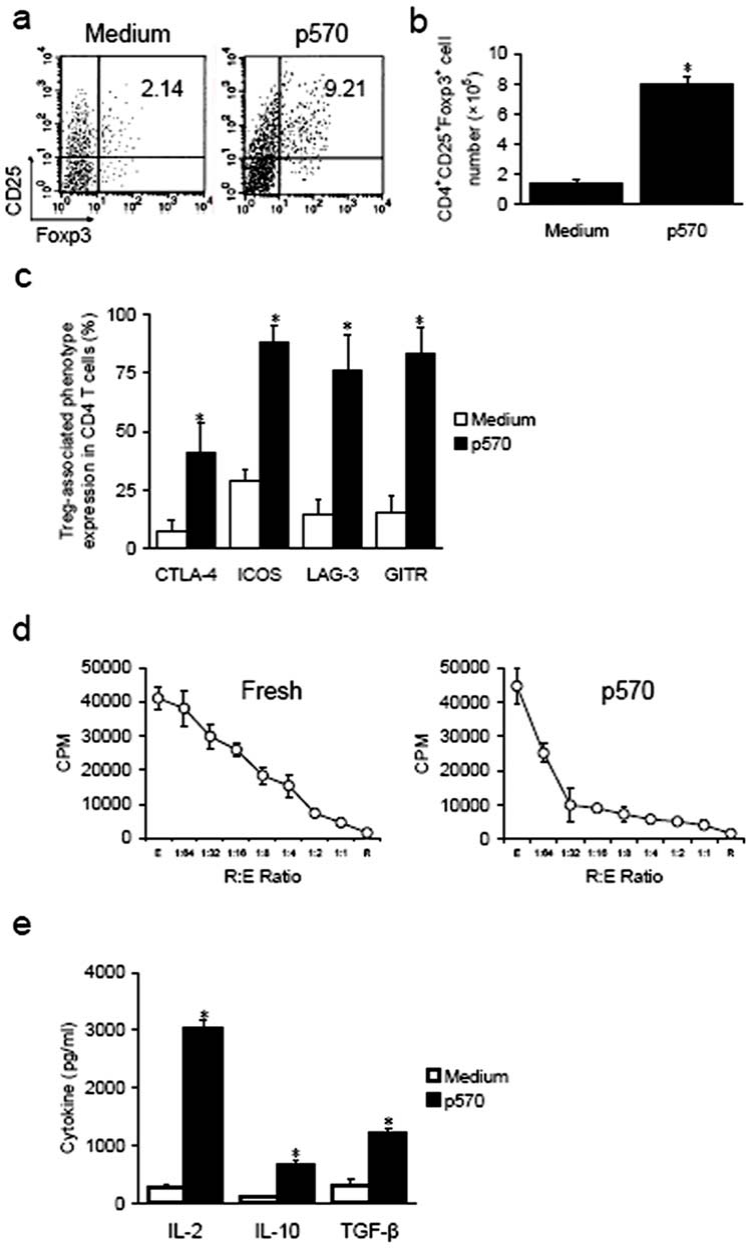
*In vitro* expansion of CD4^+^CD25^+^ T cells by p570 stimulation. Splenic lymphocytes were isolated from one NOD mice (total number of mice is 8) and cultured with synthesized p570 (50 ng/ml) for 7–10 d. Cells were collected and assayed by flow cytometry. (a) The percentage of CD25^+^Foxp3^+^ within CD4^+^cells was presented. (b) Total number of CD4^+^CD25^+^Foxp3^+^ T cells in each group after expansion. Data collected from three independent experiments are shown. (c) Expression of the Treg-related phenotype (CTLA-4, GITR, ICOS, LAG-3) in CD4 T cells in each group. (d) Fresh and p570-expanded CD4^+^CD25^+^ T cells (R) were compared for their ability to suppress proliferation of CD4^+^ effector T cells (E) under the same conditions as other peptides indicated above. (e) After incubation with p570, IL-2, IL-10 and TGF-β production of expanded CD4^+^CD25^+^ T cells was assayed by sandwich ELISA. Data shown are means±SD. *, *P*<0.05 compared with controls.

To test this hypothesis, the ability of p570 to expand CD4^+^CD25^+^ T cells was examined. Results showed that the stimulation of p570 led to a significant increase in the frequency and number of CD4^+^CD25^+^Foxp3^+^ T cells after 7–10 days culture (**Figure 9a and 9b**). Treg-associated phenotype expression, including CTLA-4, GITR, ICOS, and LAG-3, was also markedly up-regulated (**Figure 9c**). The suppressive capacity of these expanded CD4^+^CD25^+^ T cells was determined by active inhibition of the proliferation of Teffs (**Figure 9d**). CD4^+^CD25^+^ T cells expanded by p570 stimulation produced high levels of IL-2, IL-10 and TGF-β comparable to those induced by p524 stimulation (**Figure 9e**). Taken together, these data indicate that p570 was a potential epitope for Treg expansion.

P570-expanded CD4^+^CD25^+^ T cells suppress diabetes development in cotransfer models

**Figure 10:**
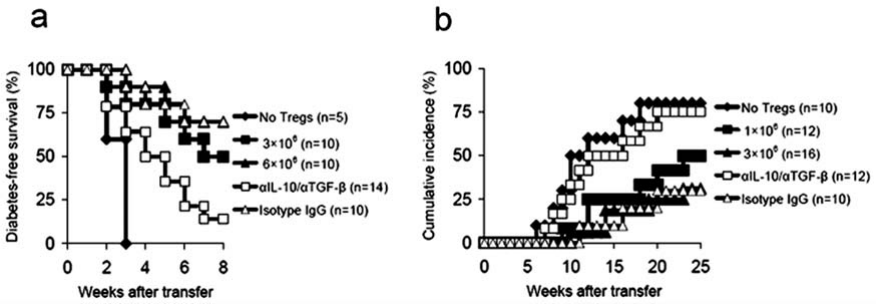
P570-expanded CD4^+^CD25^+^ T cells suppressed development of diabetes. (a) Diabetic splenocytes (1×10^7^) were co-transferred with CD4^+^CD25^+^ T cells expanded by p570 to 4–8 wk old NOD.scid mice at the numbers indicated. Control recipients were injected with diabetic splenocytes (1×10^7^) alone. In some cases, NOD.scid recipients receiving 6×10^6^ p570-expanded CD4^+^CD25^+^ T cells were injected with neutralizing antibodies to IL-10/TGF-β according to the regimens described in Materials and Methods. Blood glucose levels were monitored for up to 8 wks. Differences between diabetic spleens alone and diabetic spleens plus 3×10^6^ p570-expanded CD4^+^CD25^+^ T cells were significant (P = 0.0039), as were differences between diabetic spleens alone and diabetic spleens plus 6×10^6^ p570-expanded CD4^+^CD25^+^ T cells (P = 0.0031). (b) P570-expanded CD4^+^CD25^+^ T cells were transfused into 4–6 wk old NOD mice at the numbers indicated. Untreated littermates were used as controls. In some cases, NOD recipients receiving 3×10^6^ p524-expanded CD4^+^CD25^+^ T cells were injected with neutralizing antibodies to IL-10/TGF-β according to the regimens described in Materials and Methods. Development of diabetes was monitored over 25 wks. The difference between untreated controls and mice transfused with 1×10^6^ p570-expanded CD4^+^CD25^+^ T cells was significant (P = 0.0043), as was the difference between untreated and 3×10^6^ p570-expanded CD4^+^CD25^+^ T cells (P = 0.0025). The number of mice in each group is indicated in parentheses.

We also investigated the suppressive function of p570-expanded CD4^+^CD25^+^ T cells in two transfer models as described above. Like p524-expanded CD4^+^CD25^+^ T cells, p570-expanded CD4^+^CD25^+^ T cells actively inhibited diabetes transfer to NOD.scid mice (**Figure 10a**), and the transfer of 1×10^6^ p570-stimulated CD4^+^CD25^+^ T cells was sufficient to prevent overt autoimmunity in young NOD mice (**Figure 10b**). Again, our data indicated that p570-expanded CD4^+^CD25^+^ T cell-mediated protective effects in two models were partially due to IL-10/TGF-β secretion of these cells as blockade of IL-10/TGF-β activities in vivo dramatically abrogated the prevention from diabetes development which was mediated by p570-expanded CD4^+^CD25^+^ T cells (**Figure 10a and b**). Overall, these data suggest that p570 is also a protective peptide epitope.

## Discussion

Cell therapy for treating progressive autoimmune diseases based on in vitro expansion of Tregs has been established in the past few years [39], [42]–[43]. However, the efficiency of this strategy requires further improvement. Key issues needing to be resolved is whether the acquisition of therapeutic Tregs depends on the peptide epitope used and dissecting the relationship between the physical-chemical properties of epitopes and their function in inducing/expanding therapeutic Tregs. In the current study, we first addressed the capacity of three epitopes from the GAD_500–585_ region which were significantly different in structural properties, to expand CD4^+^CD25^+^Foxp3^+^ T cells *in vitro*. CD4^+^CD25^+^ T cells, expanded in response to stimulation with either epitopes p509, p530 or p524, exhibited excellent suppressive capacity *in vitro*. Intriguingly, when their suppression capability was tested *in vivo*, only p524-pulsed CD4^+^CD25^+^ T cells exhibited suppressive effects on diabetes development in co-transfer models.

This discrepancy between *in vivo* and *ex vivo* data appears not to result from the transfer of fewer p509 or p530-expanded CD4^+^CD25^+^ T cells compared to those expanded by p524, as even a 3–5 fold increase (e.g. 3−5×10^6^) in p509 or p530-expanded CD4^+^CD25^+^ T cells failed to inhibit transfer of diabetes (**Figure 4**). The data presented in this study indicate that this failure to inhibit transfer of diabetes may be attributed to the differential cytokine profiles of expanded CD4^+^CD25^+^ T cells. P524-expanded CD4^+^CD25^+^ T cells produced higher levels of IL-10 and TGF-β than those expanded by p509 or p530 stimulation. Furthermore, *in vivo* neutralization of IL-10/TGF-β bioactivities led to the abrogation of protective effects of p524-expanded CD4^+^CD25^+^ T cells (**Figure 4b** **and** **5b**), which is consistent with the well-accepted conception that these two categories of cytokines are recognized as important mediators of Treg immunomodulatory function [44], [45]. In addition, differences between epitope-expanded CD4^+^CD25^+^ T cells in terms of migration to or re-activation in the draining lymph nodes also need consideration. Tang et al. [32] proposed that *ex vivo*-expanded BDC2.5-specific Tregs underwent significant proliferation after transfer into NOD mice. However, expanded GAD286-specific Tregs failed to proliferate in pancreatic lymph nodes. Whether this difference in proliferation and/or migration *in vivo* is also present in p524, p509 or p530-expanded CD4^+^CD25^+^ T cells when transfused into NOD mice needs to be elucidated in further studies.

As well known, to deeply understand the biological role of the protein or peptides, the 3-D structure of them will be required. Although experimental structure determination methods are providing high-resolution structure information about a subset of the proteins, computational structure predictions will provide valuable information for the large fraction of sequences whose structures will not be determined experimentally.

Recently, studies have indicated that the biological functions of the peptide-major histocompatible complex (MHC) depend on the structural property of the peptide [46], [47]. Here, we focused on the correlation of epitope structural properties with their functions in expanding therapeutic Tregs. Quinn et al. [9] carried out experiments on peptide epitope immunization and showed that GAD524–543 consisted of at least two I-A^g7^-restricted determinants (p524 and p530, GAD530–543), with apparently distinct properties. P524-specific T cells dominate the protective response after immunization with GAD524–543, while CD4^+^ T cells arise spontaneously in young NOD mice in response to a dominant determinant found within p530. Consistent with this we have shown a difference in structural properties between p524 and p530 using molecular modeling (**Figure 6** **and** **7**). This difference may influences the conformation of MHC class II I-A^g7^ in NOD mice when bound to the corresponding peptide epitopes, as previously reported [48].

To address this issue, the patterns of p524, p530, p509 bound to I-A^g7^ were determined by molecular modeling using the structural framework of GAD peptide p207 (GAD207–220) bound to I-A^g7^ [49]. The structural properties and binding pattern to I-A^g7^ of another defined protective GAD peptide epitope, p286 (GAD286–300) [41], used here as a positive control, were also analysed. We found that the secondary structures of p524 and p286 were alpha-helices and stable, while peptides p509 and p530 were coiled and very flexible. The 3-D structure of I-A^g7^ in complex with peptides p509, p524, p530 and p286 revealed two kinds of binding mode; the binding mode of I-A^g7^ to p524 and p286 was similar, but markedly different from its binding mode with p509 and p530 (**Figure 8**). Overall, our data strongly suggest that protective peptide epitopes p524 and p286, binding to I-A^g7^, could form a unique conformation on the surface of APCs, allowing for the induction of Tregs.

To support this premise we analyzed another epitope p570, which is very similar to p524 in sequence tendency, surface electrostatic potential and interaction mode with I-A^g7^. Similar structural properties and binding patterns of p570 to I-A^g7^, to those of p524 or p286 were revealed in this analysis. The fact that p570 stimulation facilitated induction of IL-10/TGF-β-secreting CD4^+^CD25^+^ T cells indicates that p570 is a protective epitope. Therefore, while further comprehensive study is required, our present data highlight that computer modeling-based T cell-epitope study is a promising tool for function prediction of many autoimmune-related epitopes. We are currently analyzing the epitopes of another two critical autoimmune diabetes-related autoantigens (i.e. insulin and IA-2) using molecular modeling.

To our knowledge, this is the first report of molecular modeling-based analysis of T cell epitope function and prediction. Classical approaches, such as use of 15-mer overlapping synthetic peptides, are costly and time-consuming. With the help of molecular modeling, it will become possible to carry out large-scale screening of antigen/peptide targets, providing valuable structural information as well as predicting potential epitopes and their functions. Interestingly, to date, several of the GAD-specific clones isolated have been found to be pathogenic and quite a few are non-pathogenic [50], [51]. Accumulating evidence indicates that many GAD-specific responses may be protective and not diabetogenic. As a result, it is reasonable that GAD should be used preferentially as an immunogen to induce protective effects or to abrogate aggressive autoimmunity. The present study proposes two important regulatory epitopes (p524 and p570) in the region of GAD_500–585_ for expanding functional Tregs. These findings may be helpful for the design of autoimmune-related autoantigen epitopes for immune intervention and achievement of therapeutic epitope-specific Tregs.
